# Drug-induced fever: a pharmacovigilance analysis based on the FDA adverse event reporting system

**DOI:** 10.1007/s11096-026-02113-3

**Published:** 2026-04-01

**Authors:** Xiaozhi Li, Weichun Zhang, Xiaofen Wen, Danxia Lin, Xiajiong Luo

**Affiliations:** 1 Department of Medical Oncology, Cancer Hospital of Shantou University Medical College, Shantou, China; 2 Department of Pharmacy, The Second Affiliated Hospital of Shantou University Medical College, Shantou, China

**Keywords:** ADEs, Drug-induced fever, FAERS, Pharmacovigilance, Time-to-onset

## Abstract

**Introduction:**

Drug-induced fever is a common but often under-recognized adverse reaction that can complicate clinical management.

**Aim:**

This study aimed to systematically evaluate the risk of drug-induced fever using real-world pharmacovigilance data and to characterize its temporal onset patterns and clinical features.

**Method:**

A pharmacovigilance study was conducted utilizing data from the FAERS, covering reports from the first quarter of 2004 to the fourth quarter of 2024. The detection of adverse drug event signals was conducted using disproportionality analysis. The robustness of identified fever signals was assessed through sensitivity analysis. Furthermore, a time-to-onset analysis was undertaken to elucidate factors associated with the development of drug-induced fever.

**Results:**

Disproportionality analysis revealed that antineoplastic and immunomodulating agents were the most frequently associated, followed by antiinfectives for systemic use, alimentary tract and metabolism, musculo-skeletal system, nervous system, blood and blood forming organs, dermatologicals, genito urinary system and sexhormones. The top-five drugs with drug-induced fever signals by case number were rituximab (3650 cases), immunoglobulin human normal (2478 cases), zoledronic acid (2304 cases), lamotrigine (1843 cases), dabrafenib (1803 cases). Sensitivity analysis confirmed that the majority of the positive signals remained robust. Analysis of the time-to-onset revealed that drug-induced fever occurred predominantly during the early phase of treatment. Furthermore, female patients experienced an earlier onset compared to males.

**Conclusion:**

This large-scale pharmacovigilance analysis identified drugs and patient characteristics associated with drug-induced fever and revealed early-onset patterns, providing evidence to support its early recognition and monitoring in clinical practice.

**Supplementary Information:**

The online version contains supplementary material available at 10.1007/s11096-026-02113-3.

## Impact statements

- This study highlights the importance of monitoring for drug-induced fever, particularly with high-risk agents.
- Observed differences in time-to-onset by patient characteristics, such as sex, highlight the potential value of incorporating demographic factors into clinical risk assessments.
- Findings from real-world pharmacovigilance data provide evidence to support timely recognition and monitoring of drug-induced fever in clinical practice.

## Introduction

Drug-induced fever, a frequently underrecognized adverse drug reaction, is characterized by a febrile response that develops during pharmacotherapy and resolves upon discontinuation of the causative agent. Diagnosis necessitates a comprehensive clinical evaluation involving detailed medical history review, thorough physical examination, and extensive laboratory investigations to rule out other etiologies [1, 2]. Epidemiological data from the United States indicate that adverse drug reactions occur in approximately 10–15% of hospitalized patients, with drug-induced fever constituting 3–5% of all adverse drug reactions (ADRs). However, due to diagnostic challenges and significant underreporting, the true incidence remains uncertain [3]. The pathogenic mechanisms remain incompletely understood but are thought to involve hypersensitivity reactions, idiosyncratic metabolic responses, excessive pharmacological effects, disruption of thermoregulation, and direct effects related to drug administration techniques [2–4].

Drug-induced fever typically resolves spontaneously upon discontinuation of the suspected drug, unlike other types of fever.For clinicians, promptly identifying high-risk medications and patient populations is critical, as early diagnosis can shorten hospital stays, reduce morbidity, and lower unnecessary healthcare costs [5]. Current evidence identifies antibacterial agents, central nervous system-acting(CNS) drugs, antineoplastic therapies, and cardiovascular medications as the most common causes of drug-induced fever [3]. Nevertheless, current understanding of this phenomenon largely comes from isolated case reports and small case series, highlighting the need for large-scale, real-world epidemiological studies to better characterize its clinical profile.

The primary objective of this study is to systematically investigate drug-induced fever using large-scale real-world data from the FAERS database. As the pharmaceutical market expands, heightened awareness of drug-induced fever as a clinically significant reaction is crucial. Currently, there is no well-established consensus for the diagnostic criteria, epidemiology, or risk factors of drug-induced fever. This study aimed to bridge this knowledge gap by performing comprehensive analyses of real-world data to validate existing findings, identify previously unrecognized drug-fever associations, and advance a more robust clinical understanding of this condition. We aimed to identify medications most frequently associated with drug-induced fever, and assess their time to onset.

## Aim

This study aimed to systematically evaluate the risk of drug-induced fever using real-world pharmacovigilance data and to characterize its temporal onset patterns and clinical features.

## Methods

### Data source

This disproportionality analysis used the FAERS, an FDA-maintained pharmacovigilance database that systematically collects spontaneous reports of adverse drug events and medication errors from healthcare providers, consumers, and pharmaceutical manufacturers. The database primarily consists of seven datasets: patient demographic and administrative information (DEMO), drug information (DRUG), adverse events (REAC), patient outcomes (OUTC), report sources (RPSR), therapy start dates and end dates for reported drugs (THER), and indications for drug administration (INDI). Within FAERS, all adverse events are coded to the Preferred Terms (PTs) of the Medical Dictionary for Regulatory Activities (MedDRA). As the database contains only de-identified and publicly accessible patient information, this study was exempt from ethical approval and informed consent requirements.

### Data processing

**Fig. 1:**
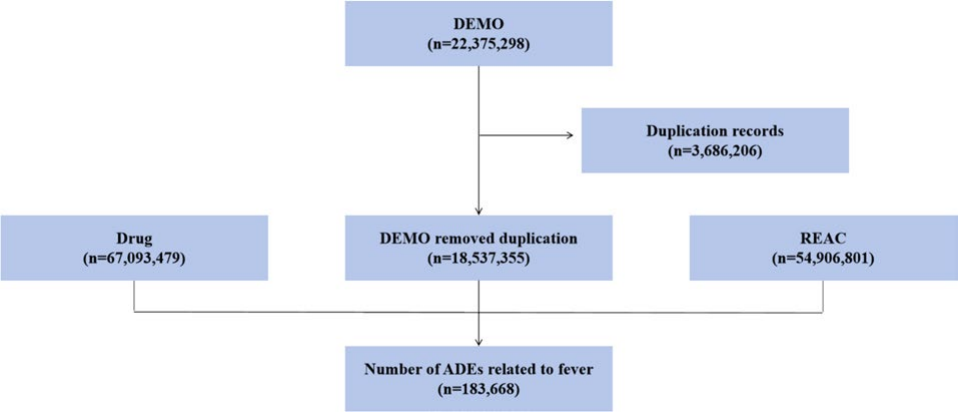
Flow chart of the study for drug-induced fever data in the FAERS database. Abbreviations: DEMO, patient demographic and administrative information; DRUG, drug information; REAC, adverse events; ADEs, Adverse Drug Events

This retrospective pharmacovigilance analysis spanned Q1 2004 through Q4 2024, in accordance with the timeframe of publicly available FAERS data. We extracted the PRIMARYID, CASEID, and FDA_DT fields from the DEMO table and sorted the records by CASEID, FDA_DT, and PRIMARYID. For each CASEID, we retained only the record with the largest FDA_DT value. In cases where multiple reports had identical CASEID and FDA_DT, the record with the largest PRIMARYID value was retained. The entire process of deduplication was based on recommendations by the FDA. Adverse events were identified by the MedDRA PT “Pyrexia” (per MedDRA guidance, temperature-related terms such as high temperature and spiking temperature are coded under PT Pyrexia), with a list of these Lowest Level Terms provided in Table S1. Reports in the FAERS database are categorized into four types based on the role of the medication: primary suspect, secondary suspect, concomitant, and interacting. To ensure accuracy, this study included only reports in which the medication was classified as the primary suspect. Subsequently, the nomenclature for drugs of different formulations was standardized to their generic names via the DrugBank database (www.drugbank.com). To enhance data quality and minimize the inclusion of erroneous reports, the study was restricted to cases reported by health professionals [6, 7]; reports from non-health professionals, such as consumers or lawyers, were excluded. To ensure transparency and reproducibility in disproportionality analysis, this pharmacovigilance study was conducted following the Reporting of a Disproportionality Analysis for Drug Safety Signal Detection Using Individual Case Safety Reports in PharmacoVigilance (READUS-PV) guidelines [8, 9]. The flow of data processing is detailed in Fig. 1.

### Disproportionality analysis

We employed four disproportionality analysis methods, including reported odds ratio (ROR) [10], proportional reporting ratio (PRR) [11], bayesian confidence propagation neural network (BCPNN) [12], and multi-item gamma poisson shrinker (MGPS) [13], to identify potential associations between drugs and drug-induced fever. Non-Bayesian approaches, including ROR and PRR, are particularly suitable for early signal detection, whereas Bayesian methods such as BCPNN and MGPS are more effective in identifying rare or sparse signals, especially when the number of reported cases is limited. A statistically significant signal was designated only when results satisfied all four algorithms' positive criteria concurrently. Detailed calculation methods and thresholds are presented in Table S2. To minimize false-positive findings, only drugs associated with fever and with a minimum of 100 reported cases were included in the analysis. We classified the detected signal drugs using the Anatomical Therapeutic Chemical (ATC) system to determine their therapeutic categories and analyze associations with adverse events. Additionally, the Summary of Product Characteristics (SPCs) from the FDA were consulted to determine whether these drugs carried any fever-related warnings.

### Sensitivity analysis

To mitigate potential confounding bias introduced by patient characteristics, logistic regression was performed on suspected drugs that had more than 100 reported cases to assess the robustness of the primary analysis. Statistically significant variables from the univariate logistic regression were included in a subsequent multivariate analysis. The multivariate logistic regression analysis was adjusted for variables including patient sex, age, weight, primary disease, and concomitant medications, yielding the adjusted reporting odds ratio (aROR) along with its 95% confidence interval (CI). This approach ensures that the observed association between the drugs and fever is not attributable to these confounding factors, thereby enabling a more rigorous and reliable assessment of the relationship between the signal drugs and fever.

### Time-to-onset analysis

The time to onset is defined as the duration between EVENT_DT (the date of adverse event onset) and START_DT (the date of treatment initiation). Reports with data entry errors, such as those in which EVENT_DT precedes START_DT, or with otherwise inaccurate date records, were excluded. The time to fever onset was characterized using the median, quartiles, and the Weibull Shape Parameter (WSP). WSP analysis characterizes the temporal distribution of reported events using the scale (α) and shape (β) parameters. When β < 1 and the upper bound of the 95% confidence interval (CI) < 1, the frequency of adverse events initially increases and then declines, corresponding to the early failure type. When β > 1 and the lower bound of the 95% CI > 1, the frequency of adverse events increases progressively over time, indicating the wear-out failure type. When β ≈ 1 and the 95% CI includes 1, the incidence of adverse events remains relatively constant throughout treatment, which is classified as the random failure type. Furthermore, disparities in onset time across sex, age groups, and drug class were assessed by comparing their cumulative distribution curves.

### Statistical analysis

In stratified analyses by sex, age group, and drug class, differences in median time to event onset were assessed using the Mann–Whitney U test and the Kruskal–Wallis test. A two-tailed *P *value < 0.01 was defined as statistically significant. All statistical analyses were conducted using R (version 4.5.0) in RStudio.

### Ethics approval

This study utilized publicly available, de-identified data from FAERS. As no patient-identifiable information was accessed, institutional ethics approval was not required.

## Results

### Descriptive analysis

Between Q1 2004 and Q4 2024, a total of 183,668 unique cases of drug-induced fever were documented in the FAERS database. Among these, female patients constituted 90,700 cases (49.4%), outnumbering male patients, who accounted for 76,921 cases (41.9%). The most affected age group was 45–65 years, representing 27.5% of all cases, followed by patients over 65 years (24.6%) and those aged 18–45 years (17.6%). The United States reported the highest number of cases (65,326, 35.6%), followed by Canada (15,188, 8.3%) and Japan (14,187, 7.7%). The most frequently reported adverse outcome was hospitalization. The annual numbers of reports from 2004 to 2024 are shown in Table 1.

**Table 1:** Clinical characteristics of patients with drug-induced fever in the FAERS databases

Characteristics	Number (%)
Number of patients	183,668
*Sex*	
Female	90,700 (49.4%)
Male	76,921 (41.9%)
Missing	16,047 (8.7%)
*AGE(year)*	
< 18	15,958(8.7%)
≥ 18, < 45	32,360(17.6%)
≥ 45, < 65	50,546(27.5%)
≥ 65	45,093(24.6%)
Missing	39,711(21.6%)
*Reported countries (Top 5)*	
United States	65,326 (35.6%)
Canada	15,188 (8.3%)
Japan	14,187 (7.7%)
France	8220 (4.5%)
Germany	7221 (3.9%)
*Outcome*	
Death	19,513 (10.6%)
Disability	1714 (0.9%)
Hospitalization	86,897 (47.3%)
Life-threatening	10,648 (5.8%)
Other	64,896 (35.3%)
*Received year*	
2004	3793 (2.1%)
2005	3717 (2.0%)
2006	3772 (2.1%)
2007	3291 (1.8%)
2008	3972 (2.2%)
2009	4524 (2.5%)
2010	5364 (2.9%)
2011	6540 (3.6%)
2012	7709 (4.2%)
2013	8044 (4.4%)
2014	8206 (4.5%)
2015	9725 (5.3%)
2016	9798 (5.3%)
2017	10,269 (5.6%)
2018	13,088 (7.1%)
2019	13,845 (7.5%)
2020	14,519 (7.9%)
2021	13,363 (7.3%)
2022	12,822 (7.0%)
2023	12,846 (7.0%)
2024	14,461 (7.9%)

### Disproportionality analysis

**Fig. 2:**
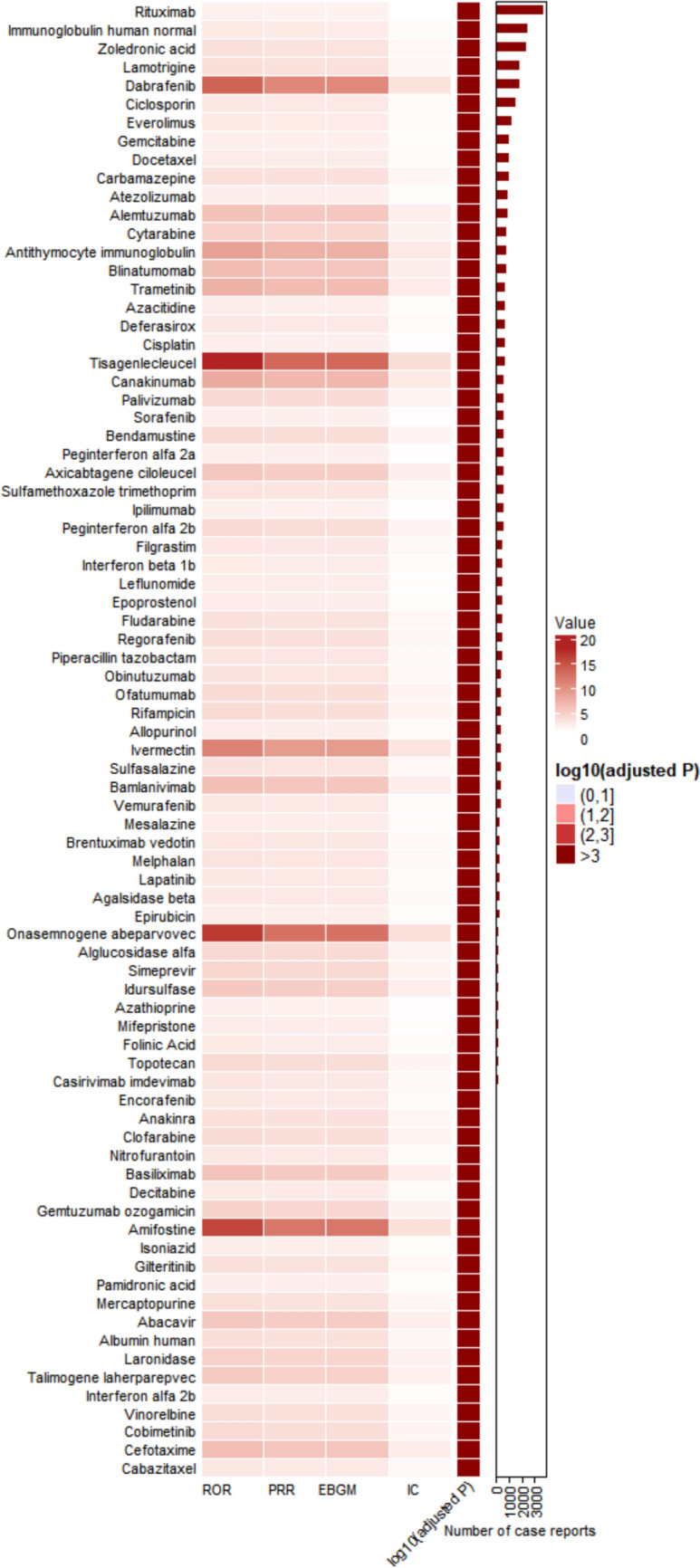
Signaling drugs associated with drug-induced fever from the FAERS Database

A total of 80 drugs meeting the criteria of having over 100 reported cases and exhibiting positive signals were identified (Fig. 2). These drugs with positive signals were categorized according to the WHO ATC Classification System (Table S3). The top five noteworthy drug categories were as follows: antineoplastic and immunomodulating agents (29,186 cases, 49 drugs), antiinfectives for systemic use (5587 cases, 11 drugs), musculo-skeletal system (1674 cases, 6 drugs), nervous system (2836 cases, 2 drugs), and alimentary tract and metabolism (635 cases, 2 drugs).

### Sensitivity analysis

**Fig. 3:**
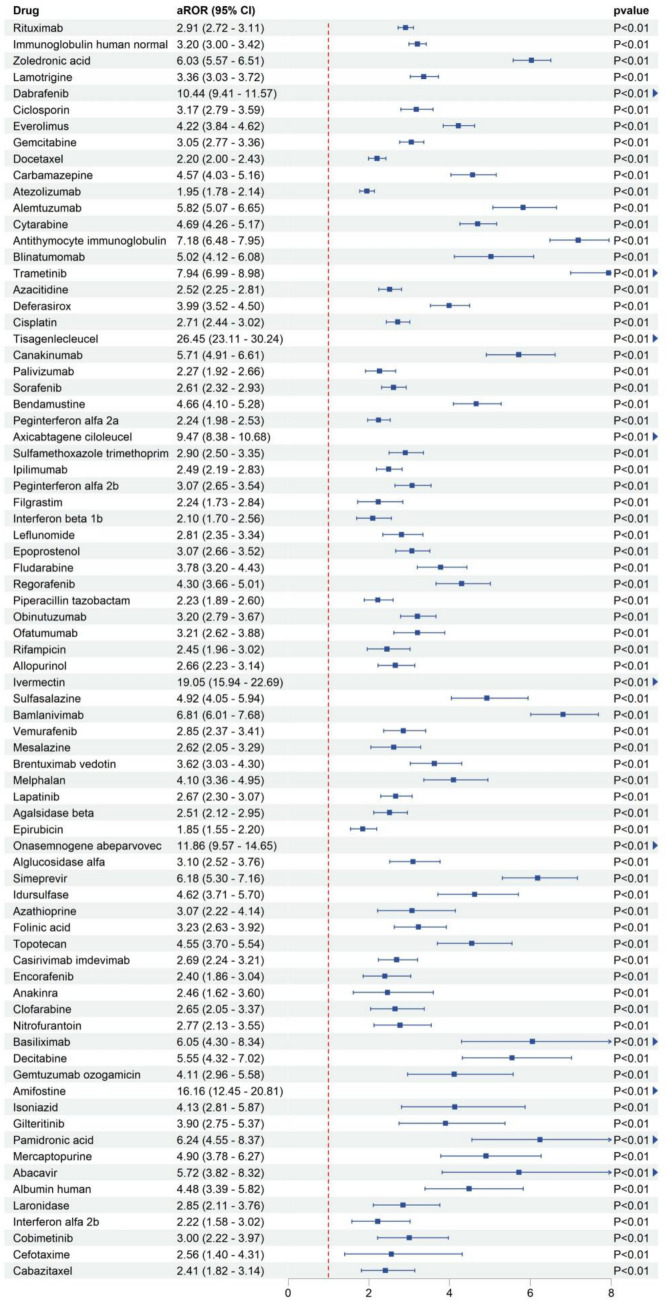
Adjusted reporting odds ratio (aROR) of drug-induced fever

In the sensitivity analysis encompassing all 80 signaled drugs, all except mifepristone, talimogene laherparepvec, and vinorelbine were confirmed to be associated with fever (Fig. 3). This finding is consistent with the results from the primary analysis.

### Time-to-onset analysis

The analysis revealed significant variations in the TTO of drug-induced fever across the evaluated medications. The shortest median TTO (0.5 days) was observed for several agents, including antithymocyte immunoglobulin (IQR: 0.5–2.5 days), human albumin (IQR: 0.5–1.5 days), casirivimab imdevimab (IQR: 0.5–0.5 days), normal human immunoglobulin (IQR: 0.5–12.5 days), simeprevir (IQR: 0.5–1.5 days), and bamlanivimab (IQR: 0.5–1.5 days). In contrast, idursulfase was associated with the longest median TTO of 476.5 days (IQR: 70.5–1371.5 days) (Table S4).

Isoniazid and ivermectin follow a random failure pattern, suggesting that drug-induced fever can occur at any time during treatment without specific temporal dependence. Mifepristone demonstrated a shape parameter (β) greater than 1, indicative of a wear-out failure pattern, in which reports of drug-induced fever increase with time. In contrast, the remaining drugs had a shape parameter (β) less than 1, consistent with an early failure pattern, where fewer cases of drug-induced fever are reported as treatment duration extends (Table S4).

**Fig. 4:**
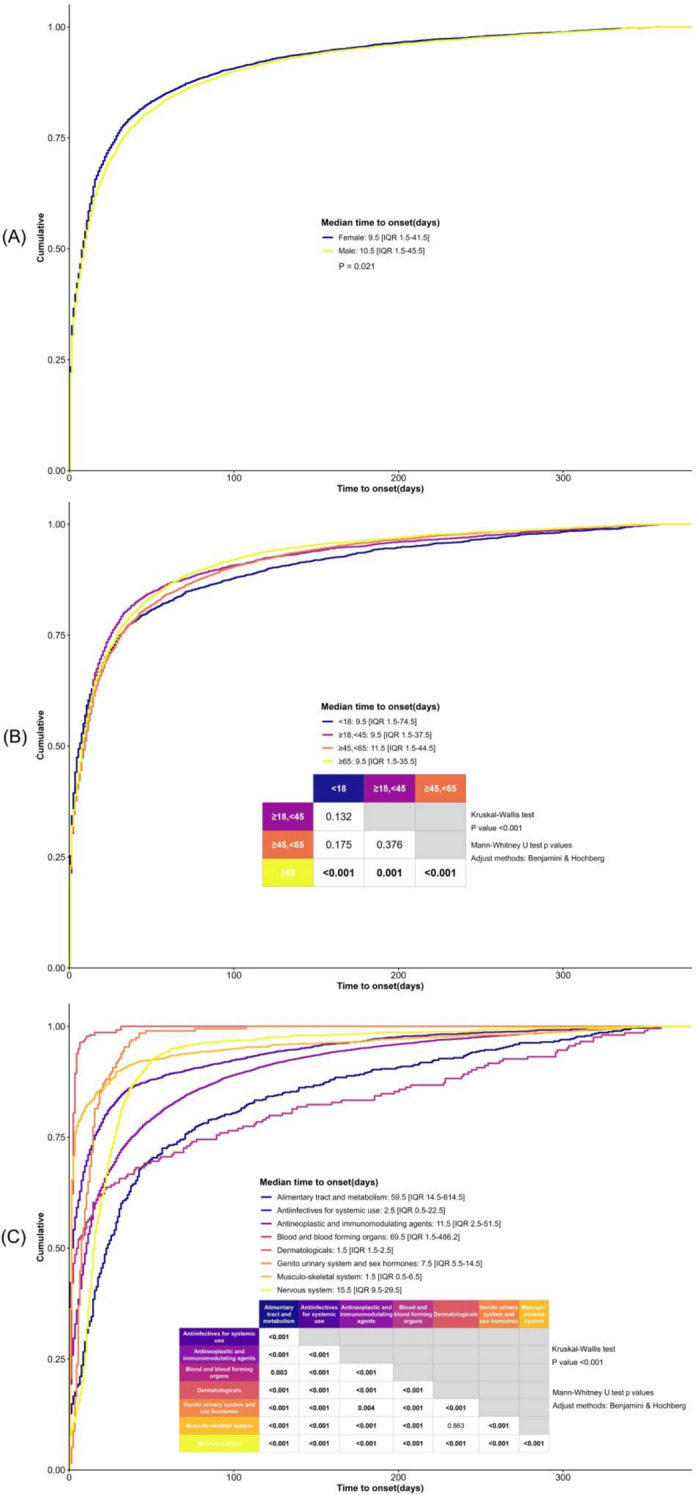
Analysis of time-to-onset of drug-induced fever. **a** Cumulative onset distribution of drug-induced fever by sex. **b** Cumulative onset distribution of drug-induced fever by age. **c** Cumulative onset distribution of drug-induced fever by drug class. Statistical analysis: Continuous variables were analyzed using the Mann–Whitney U test for two-group comparisons and the Kruskal–Wallis test for multi-group comparisons, with Benjamini–Hochberg false discovery rate (FDR) correction for multiple testing

To further investigate factors affecting the time-to-onset, we performed stratified analyses by drug class, sex, and age. The cumulative distribution curve analysis demonstrated that the median time to onset varied significantly across most drug classes, with the exception of musculo-skeletal system and dermatologicals. Regarding sex differences, the onset of drug-induced fever occurred earlier in females (median 9.5 days, IQR: 1.5–41.5 days) than in males (median 10.5 days, IQR: 1.5–45.5 days) (Fig. 4A). In addition, no significant difference in the time to drug-induced fever onset was observed among patients under 65 years of age (Fig. 4B). Analysis by drug class (Fig. 4C) revealed that dermatologicals (median 1.5 days, IQR: 1.5–2.5 days) and musculo-skeletal system drugs (median 1.5 days, IQR: 0.5–6.5 days) demonstrated the earliest onset, followed by anti-infectives for systemic use (median 2.5 days, IQR: 0.5–22.5 days), genito urinary system and sex hormones (median 7.5 days, IQR: 5.5–14.5 days), antineoplastic and immunomodulating agents (median 11.5 days, IQR: 2.5–51.5 days), and nervous system drugs (median 15.5 days, IQR: 9.5–29.5 days). In contrast, agents affecting the blood and blood forming organs showed the latest onset (median 69.5 days, IQR: 1.5–485.2 days), while alimentary tract and metabolis drugs exhibited the second-longest median time to onset (median 59.5 days, IQR: 14.5–614.5 days).

## Discussion

In this study, we performed a systematic and comprehensive analysis of drug-induced fever cases reported in the FAERS database over a 20-year period, from January 2004 to December 2024. Through quantitative signal detection methods, a total of 80 drugs meeting the threshold of over 100 reported cases and demonstrating positive signals were identified. We further explored the time-to-onset profile of drug-induced fever and conducted stratified subgroup analyses by age, sex, and therapeutic drug categories. To our knowledge, this is the first large-scale real-world analysis using the FAERS database to map the risk landscape of drug-induced fever. Our findings provide critical insights to enhance pharmacovigilance strategies, support potential updates to drug labeling, and aid clinicians in identifying high-risk medications linked to this adverse reaction.

Drug-induced fever is often missed in clinical practice because of its nonspecific presentation and absence of localized signs. When febrile episodes remain unexplained after excluding other potential causes, clinicians should consider medications commonly known to induce drug-induced fever. Evidence from clinical research, case reports, and literature reviews supports the documented association between the drug identified in this study and fever. Current evidence indicates that antibacterial agents are the most prevalent triggers, accounting for about one-third of cases, with β-lactams, sulfonamides, and minocycline notably implicated [5, 14]. Confirming this observation, a study found that 13.1% of patients on antibiotic therapy developed drug-induced fever [15]. Antiepileptic drugs constitute another significant cause, with an incidence of drug-induced fever in approximately 1 per 5000 patients [16]. Examples include carbamazepine, phenytoin, and phenobarbital [17–21]. Fever is also a common adverse effect triggered by antineoplastic agents, particularly in the period after chemotherapy [22]. The evolution of oncologic therapeutics has also highlighted fever risks associated with newer immunotherapies, particularly anti-PD-1 and anti-PD-L1 immune checkpoint inhibitors [23]. Of particular concern is the dabrafenib-trametinib combination, which induces fever in 40–60% of patients [24].

Our study revealed that antineoplastic and immunomodulating agents constituted the largest proportion (49/80), followed by antiinfectives for systemic use (11/80), alimentary tract and metabolism (6/80), musculo-skeletal system (4/80), nervous system (2/80), blood and blood forming organs (2/80), dermatologicals (1/80), genito urinary system and sexhormones (1/80). Compared with earlier reports that emphasized antiinfectives for systemic use, the current distribution demonstrates an increased share of aantineoplastic and immunomodulating agents. The associations between the most frequently implicated drugs and drug-induced fever are consistent with their known pharmacological mechanisms and with reports in the literature. For example, fever after rituximab administration typically reflects infusion-related cytokine release [25, 26], whereas fevers following intravenous normal human immunoglobulin usually represent acute reactions mediated by complement activation and/or cytokine release [27]. Zoledronic acid commonly triggers an acute-phase response within 72 h of the initial infusion that clinically resembles an influenza-like illness [28, 29]. In contrast, lamotrigine-associated fever is mainly linked to severe hypersensitivity, most notably drug reaction with eosinophilia and systemic symptoms (DRESS) [13]. Finally, fever observed during treatment of metastatic melanoma with dabrafenib is generally considered a treatment-related adverse reaction [14, 30].

Drug-induced fever can occur throughout pharmacotherapy. Current literature indicates a median onset of about 8 days following initiation, significant differences occur among different drug classes [3, 5, 22, 31–35]. For example, antibiotics typically cause fever within 1–5 weeks [32, 34, 36–38], while antineoplastic agents induce fever within 3–4 days [22], and nervous system drugs do so from 2 days to 8 weeks [33, 39, 40]. It should be noted that current evidence on drug-induced fever onset is largely derived from limited case reports or small case series, underscoring the need for more comprehensive analyses. Our findings revealed distinct onset patterns across drug categories: antineoplastic and immunomodulating agents showed a median onset of 11.5 days (IQR: 2.5–51.5 days), antiinfectives for systemic use showed 2.5 days (IQR: 0.5–22.5 days), and nervous system drugs showed 15.5 days (IQR: 9.5–29.5 days). These results not only corroborate previous studies but also provide more precise, drug-specific timelines for clinical application. The Weibull shape parameter test characterized drug-induced fever onset as an early failure pattern, with incidence decreasing over time. While most cases appear within weeks of starting a medication, clinicians should remain vigilant for delayed presentations, even with prolonged therapy. The observation indicates that close surveillance is warranted, especially during the initial treatment period.

As a diagnosis of exclusion, drug-induced fever requires consideration of a broad differential, including infections, malignancies, autoimmune diseases, and autoinflammatory syndromes [14]. Drug-induced fever should be included in the differential diagnosis when a febrile patient is otherwise in good clinical condition or demonstrates clinical improvement. Clinicians should perform a detailed review of medication history, onset characteristics, and laboratory investigations, as these often provide important clues in identifying drug-induced fever. History-taking should record all current medications, with particular attention to drug classes known to cause fever—notably antineoplastic and immunomodulatory agents, systemic anti-infectives, drugs acting on the alimentary tract and metabolism, and therapies for musculoskeletal and nervous system conditions. Withdrawal of the offending agent can facilitate a presumptive diagnosis and serves as an effective therapeutic measure for drug-induced fever [3].

Several limitations in this study deserve attention. First, adverse event reports in the FAERS database are voluntarily submitted, which may give rise to underreporting, misreporting, and reporting bias. Secondly, although our study adjusted for key potential confounders (sex, age, weight, primary disease, and concomitant medications), intrinsic reporting bias and database-specific incomplete data may still introduce residual bias. Furthermore, data from spontaneous reporting systems are suitable only for signal detection and correlational analyses and are insufficient for drawing causal inferences; consequently, the causal relationship between the reported cases and drug-induced fever remains unclear. Finally, pharmacovigilance investigations are inherently limited by underreporting, heterogeneous reporting practices and poor capture of late-onset events, which may bias disproportionality measures and hinder identification of delayed adverse events.

## Conclusion

This large-scale pharmacovigilance analysis identified drugs and patient characteristics associated with drug-induced fever and revealed early-onset patterns, providing evidence to support its early recognition and monitoring in clinical practice.

## Supplementary Information

Below is the link to the electronic supplementary material.

Supplementary file1 (XLSX 10 kb)

Supplementary file2 (DOCX 13 kb)

Supplementary file3 (XLSX 17 kb)

Supplementary file4 (XLSX 16 kb)

## Data Availability

This study is based on the FAERS databases, which are publicly accessible at https://fis.fda.gov/extensions/FPD-QDE-FAERS/FPD-QDE-FAERS.html without requiring prior applications.
